# In Vitro and In Vivo Effectiveness of Carvacrol, Thymol and Linalool against *Leishmania infantum*

**DOI:** 10.3390/molecules24112072

**Published:** 2019-05-30

**Authors:** Mohammad Reza Youssefi, Elham Moghaddas, Mohaddeseh Abouhosseini Tabari, Ali Akbar Moghadamnia, Seyed Mohammad Hosseini, Bibi Razieh Hosseini Farash, Mohammad Amin Ebrahimi, Niki Nabavi Mousavi, Abdolmajid Fata, Filippo Maggi, Riccardo Petrelli, Stefano Dall’Acqua, Giovanni Benelli, Stefania Sut

**Affiliations:** 1 Department of Veterinary Parasitology, Babol-Branch, Islamic Azad University, Babol 8415683111, Iran; youssefi929@hotmail.com; 2 Department of Parasitology and Mycology, School of Medicine, Mashhad University of Medical Sciences, Mashhad 9177899191, Iran; moghaddase@mums.ac.ir; 3 Faculty of Veterinary Medicine, Amol University of Special Modern Technologies, Amol 46131, Iran; m.abouhosseini@ausmt.ac.ir; 4 Department of Pharmacology, Babol University of Medical Sciences, Babol 8415683111, Iran; moghadamnia@gmail.com; 5 Department of Veterinary Pathology, Babol-Branch Islamic Azad University, Babol 8415683111, Iran; dr_hosseini2323@yahoo.com; 6 Research Center of Skin Diseases and Cutaneous Leishmaniasis, School of Medicine, Mashhad University of Medical Sciences, Mashhad 9137913316, Iran; HoseiniFR@mums.ac.ir; 7 Young Researcher and Elite Club, Islamic Azad University, Babol-Branch, Babol 8415683111, Iran; mabrahimi@rocketmail.com (M.A.E.); nnabavi@yahoo.com (N.N.M.); 8 School of Pharmacy, University of Camerino, 62032 Camerino, Italy; filippo.maggi@unicam.it (F.M.); riccardo.petrelli@unicam.it (R.P.); 9 Department of Pharmaceutical and Pharmacological Sciences, University of Padova, 35121 Padova, Italy; 10 Department of Agriculture, Food and Environment, University of Pisa, via del Borghetto 80, 56124 Pisa, Italy; giovanni.benelli@unipi.it; 11 Department of Agronomy, Food, Natural Resources, Animals and Environment (DAFNAE), University of Padova, 35020 Legnaro, Italy; stefania.sut@unipd.it

**Keywords:** insect vectors, neglected tropical diseases, protozoan parasites, sandflies, visceral leishmaniasis

## Abstract

Background: One of the most important causative agents of visceral leishmaniasis (VL) is *Leishmania infantum*, which is mainly spread by *Phlebotomus* and *Lutzomyia* sandflies in the Old and New World, respectively. Novel and effective drugs to manage this neglected vector-borne disease are urgently required. In this study, we evaluated the toxicity of carvacrol, thymol and linalool, three common essential oil constituents, on amastigotes and promastigotes of *L. infantum*. *Methods*: in vitro experiments were performed by 24 h MTT assay. Carvacrol, thymol and linalool at concentrations ranging from 1.3 to 10 μg/mL were tested on promastigotes of *L. infantum*. For in vivo test, two groups of hamsters (*Mesocricetus auratus*) received 100 mg/kg of body weight/day of carvacrol and thymol as intraperitoneal injection on day 7 post-infection, followed by a 48 h later injection. The third group was treated with the glucantime as standard drug (500 mg/kg) and the last group (control) just received normal saline. On the 16th day, the number of parasites and histopathological changes in liver and spleen were investigated. Results: 24 h MTT assay showed promising antileishmanial activity of thymol and carvacrol, with IC_50_ values of 7.2 (48 μM) and 9.8 μg/mL (65 μM), respectively. Linalool at all concentrations did not affect *L. infantum* promastigote viability. In vivo toxicity data of carvacrol and thymol showed that the former at 100 mg/kg was the safest and most effective treatment with little side effects on the liver. Conclusions: Overall, thymol and carvacrol are highly promising candidates for the development of effective and safe drugs in the fight against VL.

## 1. Introduction

Visceral leishmaniasis (VL) is an important neglected vector-borne tropical disease mostly affecting poor and marginalized populations worldwide [1]. VL chiefly involves an infection of the reticuloendothelial system diagnosed by observation of *Leishmania* amastigotes in the liver, spleen and bone marrow [2]. Although the number of VL cases is estimated about 300,000 annually, this number is increasing worldwide due to the lack of efficient vaccines, difficulties in insect vector control, and resistance of some *Leishmania* species to currently available drugs. VL can be fatal if untreated, with rates of mortality reported to be from 20,000 to 30,000 individuals per year [3,4]. One of the most important causative agents of VL is *Leishmania infantum,* which is spread mainly in the Mediterranean area and tropical regions of South America (with a special focus on Brazil). This parasite is vectored by female sandflies, particularly species and subspecies of *Phlebotomus* in the Old World and *Lutzomyia* in the New World. Notably, *L. infantum* is often silent in infected patients and can be diagnosed by immunological and molecular methods; laboratory diagnosis of zoonotic VL needs to be improved [5].

At present, limited chemotherapy agents are available, including old drugs or new formulations of the previous ones (antimonials and amphotericin B), which are not ideal for the treatment of VL. Toxicity, resistance, high cost and long-term treatment with chemotherapeutic agents are pushing researchers to search for natural alternatives to conventional anti-leishmanial drugs [6,7,8].

Over the centuries, a wide number of essential oil-bearing plants has been studied as a source of therapeutics for the treatment of various parasites [9,10]. Although plant-borne compounds could be appropriate alternatives to chemical drugs for several diseases, it is necessary to find standard and safe preparations before recommendation for further clinical uses. Monoterpenes are fundamental constituents of essential oils and most of them are recognized as antimicrobial substances effective against infective bacteria, fungi, viruses and even parasites [11]. Thymol and carvacrol are phenolic monoterpenes obtained from essential oils of several medicinal and aromatic plants, namely oregano (*Origanum vulgare* L.) [12], thyme (*Thymus vulgaris* L., *T. daenensis* Celak) [13], savory (*Satureja montana* L.) [14], ajowain (*Trachyspermum ammi* (L.) Sprague) [15] and *Oliveria decumbens* Vent. [16]. Linalool is a linear monoterpene alcohol found in the essential oils of *Cinnamosma madagascariensis* [17], coriander (*Coriandrum sativum* L.) [18], ligurian yarrow (*Achillea ligustica* All.) [19] and lavender (*Lavandula angustifolia* Mill.) [20]. Results of previous studies have indicated that the abovementioned monoterpenoids have shown remarkable bioactivity against *Giardia*, *Entamoeba histolytica*, *Trypanosoma cruzi* and some species of *Leishmania* [21]. Thus, due to their availability from many natural sources, and due to their low toxicity as they are considered GRAS, these compounds can be considered as significant starting points for the development of new treatments against parasites.

In this framework, the present study aimed to evaluate the efficacy of these three monoterpenoids against promastigotes and amastigotes of *L. infantum*. For the purpose, an in vitro growth inhibition assessment was performed by MTT assay on *L. infantum* promastigotes. To assess the effectiveness of such compounds in vivo evaluation test was also performed using hamsters. Animals were treated with 100 mg/kg bw of these monoterpenes on day 7 post-infection intraperitoneally. Findings of this study may provide new insights into the possible application of these natural substances to treat VL with limited side-effects on human organs.

## 2. Results

### 2.1. In Vitro Anti-leishmanial Activity

**Figure 1:**
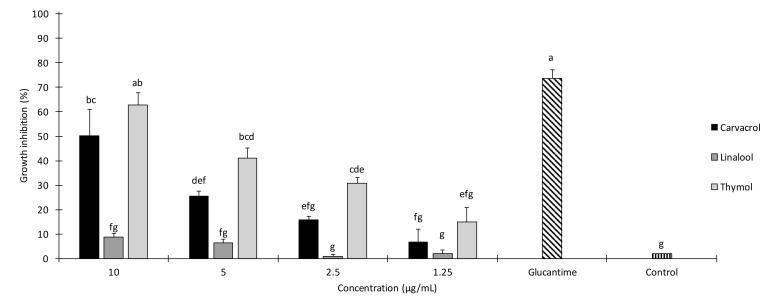
In vitro growth inhibition of *Leishmania infantum* promastigotes exposed to different concentrations of carvacrol, thymol and linalool in MTT assay lasting 24 h. Values are means ± SD; above each column, different letters indicate significant differences among treatments (ANOVA followed by Tukey’s HSD test, *p* < 0.05).

In the current study, the effects of 1.3, 2.5, 5 and 10 μg/mL of carvacrol, thymol and linalool were evaluated under in vitro conditions on promastigotes of *L. infantum*. Glucantime at 45 µg/mL was tested as positive control. Results of 24 h growth inhibition of these monoterpenes are provided in Figure 1. A significant effect of the treatment (*F_13,28_* = 26.401; *p* < 0.001) was detected. Thymol at 10, 5 and 2.5 μg/mL was highly effective against *L. infantum* in MTT-24 h over the control (*p* < 0.05). Also, carvacrol at 10 and 5 μg/mL showed significant growth inhibition if compared to the control (*p* < 0.05). Growth inhibition rates achieved by thymol and carvacrol at 10 μg/mL were significantly higher in comparison with the other treatments (*p* < 0.05), except for the thymol at 5 μg/mL (*p* > 0.05). Notably, the efficacy of thymol tested at 10 μg/mL did not significantly differ to that of the standard drug glucantime tested at 45 µg/mL (Figure 1).

Promastigotes were also exposed to different concentrations of linalool, which did not significantly affect their growth, if compared to the control (*p* > 0.05).

Results of probit analysis of the toxicity of carvacrol and thymol are provided in Table 1. Among the tested monoterpenes, thymol and carvacrol, with IC_50(90)_ values of 7.2 μg/mL (16.4 μg/mL) and 9.8 μg/mL (18.2 μg/mL), respectively, showed promising growth inhibition on *L. infantum* promastigotes.

### 2.2. Histopathological Results

**Figure 2:**
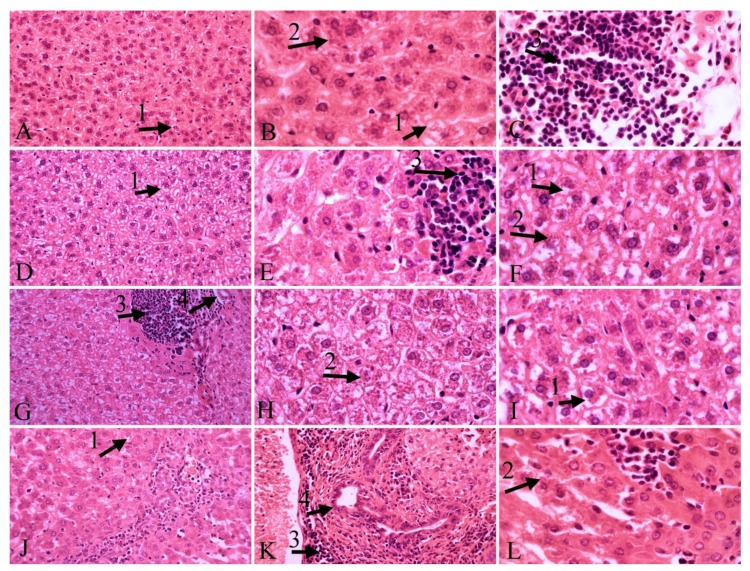
Liver tissue. **A**, **B**, **C**: control group; **D**, **E**, **F**: carvacrol group; **G**, **H**, **I**: glucantime group; **J**, **K**, **L**: thymol group; vacuolar degeneration (1), parasites in hepatocytes (2), inflammatory cells infiltration, hyperemia (3), bill duct hyperplasia (4); H&E staining, 40×, 100× magnification.

Histopathological findings in liver tissue outlined the presence of *L. infantum* parasites in hepatocytes, hyperemia, necrosis, inflammatory cells infiltration, vacuolar degeneration and Kupffer cell hyperplasia (Figure 2). Overall, inflammatory cell infiltration in the carvacrol- and glucantime-treated groups decreased when compared to the control group. Notably, these changes did not occur in the thymol-treated group. Moderate hyperemia was observed in the carvacrol- and thymol-treated groups while mild hyperemia was noted in the glucantime-treated group, if compared to the control group. The presence of *L. infantum* in the liver was significantly reduced in the thymol-treated group. Hyperplasia of the bile duct was moderate in the thymol-treated group, and mild in the glucantime-treated group. The highest degree of vacuolar degeneration was observed in the glucantime-treated group and then in the carvacrol-treated group, and mildly in the other two groups. Necrosis decreased in the thymol-treated group when compared with the other groups. Moderate hyperplasia of Kupffer cell was observed in all groups (Table 2).

**Figure 3:**
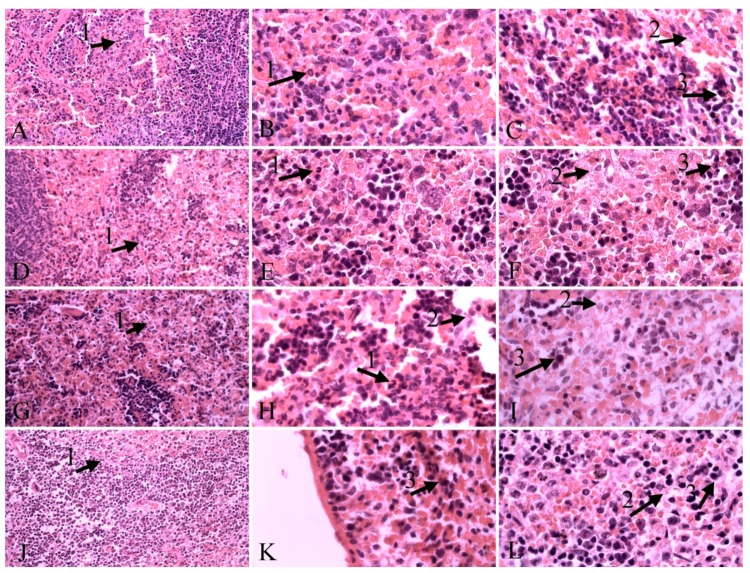
Spleen tissue. **A**, **B**, **C**: control group; **D**, **E**, **F**: carvacrol group; **G**, **H**, **I**: glucantime group; **J**, **K**, **L**: thymol group; (1), parasites in spleen cell (2), hemosiderin (3), H&E staining, 40×, 100× magnification.

Histopathological findings in spleen tissue included the presence of *L. infantum* in spleen cell, necrosis, neutrophil infiltration and hemosiderosis (Figure 3). The presence of parasites was moderate in the carvacrol-treated group, mild in the other groups. Mild necrosis was observed in all groups. Moderate presence of hemosiderin was observed in the thymol-treated group, while mild was in the other groups. Neutrophil infiltration was moderate in the glucantime-treated and control group, and mild in the other groups (Table 3).

## 3. Discussion

Carvacrol, thymol, and linalool are monoterpenoids that have been widely used for bactericidal, antiviral, fungicidal, antiparasitic, insecticidal and acaricidal purposes [22,23,24,25,26,27,28,29]. For instance, they have been reported to be effective against *T. cruzi*, as well as against the bee ectoparasitic mite *Varroa destructor* Anderson & Trueman (Acari: Varroidae) [30,31,32]. Carvacrol and thymol showed significant acaricidal activity against other mite species [33,34]. Monoterpenes are known to cause structural and functional damage of the cell membrane. Being highly lipophilic, monoterpenes may be easily absorbed by the cell membrane, thus causing destabilization of the phospholipid bilayer. They can alter the permeability of outer and inner mitochondrial membranes of eukaryotic cells, leading to apoptotic effects [35]. They may also inhibit enzymes involved in the protozoal metabolism, such as the ubiquitous enzyme DiHydroFolate Reductase (DHFR), which catalyzes the NADPH-dependent reduction of dihydrofolate to tetrahydrofolate, a precursor of cofactors required for the biosynthesis of purines, dTTP and several amino acids. Its inhibition results in a depletion of the folate pools, leading to arrest of cell proliferation and cell death. In addition, they interact directly on the synthesis and activity of ATPase, and increase the overall permeability of the cytoplasmic membrane leading to induced cell death by processes associated to the loss of osmoregulation (e.g., leakage of ATP, potassium and phosphate ions from the parasite). These activities have been detailed in various bacteria species, while little is known about the mechanism(s) of action against *Leishmania* parasites [36,37]. Therefore, further research on this issue is still needed.

The leishmanicidal activity of carvacrol and thymol against promastigotes of *L. chagasi* was outlined by Oliveira et al. [38]. Therefore, De Medeiros et al. [39] focused on the bioactivity of the essential oil from *Lippia sidoides* containing high levels of these compounds against *L. amanuensis* promastigotes. In both studies, remarkable morphological changes in the parasite have been observed and thymol was reported as the likely most active ingredient of this essential oil [38,39]. In the present study, thymol at 10 μg/mL caused the highest growth inhibition on *L. infantum* promastigotes, if compared to the control group in MTT-24 h, with a performance comparable with the standard drug glucantime at 45 μg/mL. Several studies demonstrated that the thyme essential oil is one of the most potent essential oils in terms of antimicrobial and anti-parasitic properties [22]. Also, it is effective against epimastigotes, trypomastigotes and amastigotes of *T. cruzi* [32]. On the other hand, it has also been reported that carvacrol-rich essential oils were more effective than thymol-rich oils on *L. chagasi*, and carvacrol showed lower IC_50_ values than thymol [40]. Despite the lower IC_50_ value of thymol in comparison to carvacrol (7.2 vs. 9.8 μg/mL) in our study, there is an overlap in 95% confidence limit of IC_50_ values. Therefore, this difference is not significant. Morales et al. [41] showed that carvacrol at 6.25 μg/mL has 100% inhibitory effect on *L. infantum* under in vitro conditions. However, in the present study carvacrol showed a slightly higher IC_50_ (9.8 μg/mL, 65 μM)) on *L. infantum*. In another study thymol was ineffective against amastigote of *L. chagasi*, while it was quite effective on intracellular forms of *L. (Viannia) panamensis* [42]. This variability in the efficacy of thymol may be due to different susceptibility of the tested *Leishmania* species/strains as well as to the viability assay methods used.

Besides in vitro experiments, herein we performed an in vivo evaluation of the two most effective compounds on *L. infantum* on hamsters, to shed light on the practical perspectives for real-world use of both molecules. In vivo antileishmanial activity indicated that thymol led to decrease in number of liver amastigotes in histopathological samples in comparison with the control group, while the thymol antileishmanial effect on amastigotes of the spleen was the same as that of the other compounds tested in the present study. Kupffer’s cell hyperplasia is a consequence of the phagocytic activity of these cells. The histological study of the liver showed no differences in intensity of Kupffer’s cell hyperplasia in all infected hamsters. The presence of macrophages and lymphocytes containing the parasite causes an inflammatory process in the liver infected by *L. infantum* and affects the portal tracts and inside the lobule [43]. These alterations in hepatic tissue of animals with VL have been described as inflammatory cell infiltrates [44,45]. In this investigation, the highest grade of inflammatory infiltrates in liver was observed in the control group as well as in infected animals treated with thymol, whereas hamsters treated with carvacrol and glucantime showed a reduction in the number of inflammatory cells. Although a mild degree of neutrophil infiltrations was observed in spleen after treatment with carvacrol and thymol, the untreated infected animals and the group treated with glucantime presented a moderate degree of neutrophil infiltrations. Hyperemia was observed in liver tissue of all infected groups with a higher frequency in animals treated with thymol. This process is of vital importance as a response to VL infection.

Bile duct hyperplasia may be the consequence of a toxic insult when accompanied by inflammatory cells. In this study, except control hamsters and the group treated with carvacrol, a moderate and mild bile duct hyperplasia were observed in infected treated animals with thymol and glucantime, respectively. Thus, thymol induced more specific hemosiderin deposition in the spleen compared with the other groups.

Vacuolar degeneration is a pathological change resulting in a response to nonlethal injuries [46]. The histology of liver in the present study indicated that infected animals, those treated with thymol and untreated ones had a mild hepatocyte vacuolar degeneration compared with animals treated with glucantime and carvacrol with intense and moderate hydropic change. Moreover, a mild necrotic liver tissue was observed in the infected group treated with thymol, whereas a moderate necrosis of liver was found in other infected animals. These findings demonstrated that thymol was the safest treatment with lower side effects on the liver in comparison with the other tested compounds.

## 4. Materials and Methods

### 4.1. Chemicals

Carvacrol 98% (CAS number: 499-75-2), linalool 97% (78-70-6) and thymol 98% (89-83-8) were purchased from Sigma Aldrich (Steinheim am Albuch, Germany). Serial dilutions of compounds in 1% dimethyl sulfoxide (DMSO) were prepared at a concentration of 1.25, 2.5, 5 and 10 μg/mL. Glucantime (meglumine antimoniate) was obtained from Sanofi Aventis (Paris, France).

### 4.2. Parasite Culture

Promastigotes of *L. infantum* (KT201383 strain) were maintained at 26 °C in RPMI-1640 medium supplemented with 10% bovine serum (FBS), 100 μg of streptomycin/mL, and 100 U of penicillin/mL, passaged every 3 or 4 days. Promastigotes of *L. infantum* were incubated at 26 °C for 48 h. The parasites were not used for more than three in vitro passages. A positive control (glucantime: 45 µg/mL) and a negative control (DMSO plus promastigotes) were also included. Promastigotes were diluted at a concentration of 1.0 × 10^6^ per mL of cultivation medium, then carvacrol, thymol and linalool at various concentrations were added to the experimental culture.

### 4.3. In Vitro Experiments

A MTT [3-4-5-dimethyl-2-thiazolyl)-2,5-diphenyl-2*H*-tetrazolium bromide] assay was performed to determine the growth inhibition of promastigotes 24 h after treatment with carvacrol, thymol and linalool, each of them tested at 1.25, 2.5, 5 or 10 μg/mL. Glucantime at 45 µg/mL was tested as positive control. 1% DMSO was used as negative control. MTT (1 mg/mL, pH 7.4) was added in each sample and incubated overnight in the dark at 25 °C. Afterward, isopropanol 50% and 10% sodium dodecyl sulphate (SDS) were added and incubated at 37 °C for 5 h. Finally, all samples were read at 540 nm in a microplate reader [47]. All experiments were performed in triplicate.

### 4.4. In Vivo Experiments

The in vivo study was conducted on adult male outbred golden hamsters (*Mesocricetus auratus*, mean weight 100 g) maintained in the standard condition, experimentally infected with *L. infantum*. 1 × 10^6^ promastigotes from the stationary phase of culture in RPMI1640 medium were inoculated intraperitoneally into the hamsters (four groups, four animals for each group) [42]. On day 7 post-infection, two groups received 100 mg/kg b.w. of carvacrol and thymol followed by an injection 48 h later. The third group was treated with the standard drug glucantime at 500 mg/kg and the last group left untreated and received just 1% DMSO in normal saline. All the treatments were administered intraperitoneally at the volume of 1 mL. On 16th day post-infection, all hamsters were sacrificed and tissue samples were collected. The animal study was approved by the Animal Care and Use Committee of the Medical University of Mashhad (letter ID 941107) and complied with the Principles of Laboratory Animal Care (NIH Publication no. 85–23, revised 1996).

### 4.5. Histopathological Examination

The collected liver and spleen samples were fixed in 10% buffered formalin, and then routinely processed for histopathological examination, cut into 5 μm thickness sections using a microtome (Leica, RM2235, Wetzlar, Germany). Finally, the sections were stained with hematoxylin and eosin (H&E) and viewed under light microscope (CX31- OLYMPUS, Tokyo, Japan). A TucsenTrue Chrome Metrics camera (Tucsen Photonics Co., Ltd., Fuzhou, China) and ISCapture software (Tucsen Photonics Co., Ltd., Fuzhou, China) were used for histopathological evaluation. Histopathological alteration was recorded and graded as (-) none, (+) mild, (++) moderate, and (+++) severe changes [48].

### 4.6. Statistical Analysis

Data were analyzed using SPSS software version 16 (SPSS Inc., Chicago, IL, USA): 50 and 90% inhibitory concentrations (IC_50_ and IC_90_) were calculated by probit analysis. Furthermore, statistical differences among in vitro treatments were evaluated on arcsine-transformed data by ANOVA followed by Tukey’HSD (JMP 9); *p*-values < 0.05 were considered statistically significant.

## 5. Conclusions

Overall, our research adds useful knowledge on the efficacy of three selected monoterpenes, i.e., carvacrol, thymol and linalool, against *L. infantum*, since very few data are available on this issue till now. In detail, this study demonstrated that the cyclic compounds are more active than the one presenting an open structure (linalool). Furthermore, thymol had a stronger inhibitory effect on the in vitro and in vivo growth of *L. infantum* parasites compared to the structurally related carvacrol. The significant anti-leishmanial activity of thymol described here represented an exciting advance in the search for natural drugs to be used against *Leishmania* species. Notably, the in vivo activity of thymol was very promising, stressing its potential value for real-world uses and the need of further studies to shed light on its mechanism(s) of action. Furthermore, the potential synergistic effects of blends co-formulated with phytochemicals including thymol, alone or in combination with standard drugs (e.g., glucantime, amphotericin B) [49], can also be assessed to improve efficacy in leishmaniasis treatment and to reduce the emergence of drug resistance and side effects [50].

## Figures and Tables

**Table 1:** Probit analysis showing the efficacy of carvacrol and thymol against *Leishmania infantum* promastigotes.

Treatment	Concentration (µg/mL)	Growth Inhibition after 24 h (%) ± SE ^a^	IC_50_ (µg/mL) (95% LCL–UCL)	*χ* ^2 b^
Carvacrol	10	50.3 ± 10.7	9.8 (65 μM) (8.51–11.7)	1.29 *n*.*s*.
	5	25.6 ± 2.4		
	2.5	15.76 ± 1.6		
	1.25	6.66 ± 5.2		
Thymol	10	62.76 ± 5.09	7.22 (48 μM) (6.22–8.62)	3.37 *n*.*s*.
	5	41.13 ± 4.02		
	2.5	30.83 ± 2.24		
	1.25	14.93 ± 5.92		

SE standard error, LCL 95% lower confidence limit, UCL 95% upper confidence limit, *n*.*s*. not significant (*p* ˃ 0.05). ^a^ values are mean ± SE of three replicates. ^b^ Chi-square value.

**Table 2:** Histological alteration of hamster livers in the groups treated with carvacrol, thymol and glucantime in comparison with the control group.

	Necrosis	Infiltration of Inflammatory Cells	Hyperemia	Vacuolar Degeneration of the Liver	Kupffer’s Cell Hyperplasia	Bile Duct Hyperplasia	Presence of Parasite
Control	++	+++	-	+	++	-	+++
Carvacrol	++	++	++	++	++	-	+++
Thymol	+	+++	++	+	++	++	+
Glucantime	++	++	+	+++	++	+	+++

(-) none, (+) mild, (++) moderate, and (+++) severe.

**Table 3:** Histological alteration of hamster spleens in the groups treated with carvacrol, thymol, and glucantime in comparison with the control group.

	Necrosis	Neutrophil Infiltration	Presence of Hemosiderin	Presence of the Parasite
Control	+	++	+	++
Carvacrol	+	+	+	+
Thymol	+	+	++	+
Glucantime	+	++	+	+

(+) mild, (++) moderate.
